# Hypothermic and cryogenic preservation of tissue‐engineered human bone

**DOI:** 10.1111/nyas.14264

**Published:** 2019-10-31

**Authors:** Edmund Tam, Madison McGrath, Martina Sladkova, Athbah AlManaie, Anaam Alostaad, Giuseppe Maria de Peppo

**Affiliations:** ^1^ The New York Stem Cell Foundation Research Institute New York New York

**Keywords:** bone engineering, scaffolds, stem cells, regenerative medicine, tissue banking, tissue preservation

## Abstract

To foster translation and commercialization of tissue‐engineered products, preservation methods that do not significantly compromise tissue properties need to be designed and tested. Robust preservation methods will enable the distribution of tissues to third parties for research or transplantation, as well as banking of off‐the‐shelf products. We recently engineered bone grafts from induced pluripotent stem cells and devised strategies to facilitate a tissue‐engineering approach to segmental bone defect therapy. In this study, we tested the effects of two potential preservation methods on the survival, quality, and function of tissue‐engineered human bone. Engineered bone grafts were cultured for 5 weeks in an osteogenic environment and then stored in phosphate‐buffered saline (PBS) solution at 4 °C or in Synth‐a‐Freeze™ at −80 °C. After 48 h, samples were warmed up in a water bath at 37 °C, incubated in osteogenic medium, and analyzed 1 and 24 h after revitalization. The results show that while storage in Synth‐a‐Freeze at −80 °C results in cell death and structural alteration of the extracellular matrix, hypothermic storage in PBS does not significantly affect tissue viability and integrity. This study supports the use of short‐term hypothermic storage for preservation and distribution of high‐quality tissue‐engineered bone grafts for research and future clinical applications.

## Graphical abstract

To allow distribution and banking of tissue‐engineered products, preservation methods that do not compromise tissue properties are needed. This study compared the effects of a hypothermic and a cryogenic preservation method on the survival, quality, and function of tissue‐engineered human bone and it found that the hypothermic method is better for the preservation and distribution of high‐quality tissue‐engineered bone grafts.

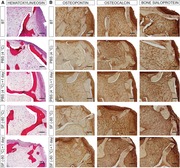

## Introduction

Tissue engineering holds the promise to revolutionize medical practice by addressing diseases and physical injuries that account for about half of the annual U.S. healthcare costs.^1^, ^2^ However, to facilitate transition from bench to bedside and commercialization of tissue‐engineered products, key manufacturing issues must be addressed. In particular, the ability to successfully preserve engineered tissues is crucial to enable banking and/or distribution of these products. The goal of preservation is to slow cellular functions to a hypometabolic state while maintaining tissue viability as well as extracellular matrix integrity and composition. Cells, tissues, and organs have been preserved for short and long periods of time at hypothermic or cryogenic temperatures^3^, ^4^, ^5^, ^6^, ^7^, ^8^, ^9^ using special media and cryoprotectants that mitigate the biophysical effects associated with the cooling, freezing, and thawing process.^10^, ^11^ Hypothermic storage is a relatively inexpensive preservation method based on the principle that biochemical events slow down at low temperatures, thus reducing the accumulation of molecular damage.^12^ At hypothermic temperature, however, the biochemical events are not completely suppressed, so damage to cells and tissues occurs. Hypothermic preservation can result in cell injury via membrane pump inactivation, disruption of calcium homeostasis, cell swelling, and free radical–induced apoptosis.^13^, ^14^, ^15^, ^16^, ^17^, ^18^, ^19^ For this reason, hypothermic preservation is generally used to prolong the shelf life of biological material only for short‐term periods during distribution and delivery. For long‐term storage and banking, cryogenic preservation is required. At very low temperatures, the metabolic activity of cells nearly arrests, and cryoinjuries are caused by the formation of ice crystals inside and outside the cells, osmotic imbalance,^20^, ^21^ and the toxicity of cryoprotectants.^22^ The biophysical effects of preservation are cell and tissue specific, and typically stronger for native and engineered tissues and organs compared with individual cells due to their higher biological complexity, poor heat transfer, and limited diffusion of cryoprotectants.^23^, ^24^ Yet, it remains unknown what the precise effects are when storing tissues engineered from induced pluripotent stem cells (iPSCs) at hypothermic and cryogenic temperatures. Human iPSCs can be derived for every patient in virtually unlimited numbers, and represent a single cell source with the ability to differentiate into all of the specialized cells constituting the bone tissue.^25^ We recently engineered bone grafts by culturing human iPSC‐derived mesenchymal progenitors onto biomimetic scaffolds in bioreactors.^26^, ^27^, ^28^, ^29^ In the present study, we asked whether these grafts could be preserved to allow distribution and banking, and studied the effects of two preservation methods on the tissue quality. Tissue‐engineered human bone grafts were stored either in phosphate‐buffered saline (PBS) solution at 4 °C or in the commercially available cryopreservation medium Synth‐a‐Freeze™ at −80 °C. After 48 h, samples were warmed up in a water bath at 37 °C, incubated in osteogenic medium, and analyzed 1 and 24 h after revitalization to take into consideration possible delays in storage‐induced cell death and tissue damage. Tissue viability was estimated via live/dead fluorescence assay and immunostaining of the apoptotic protein caspase‐3, and tissue composition was studied via immunostaining of the bone noncollagenous glycoproteins osteopontin, osteocalcin, and bone sialoprotein, while tissue function was evaluated by measuring the level of expression of the osteogenic genes, the runt‐related transcription factor 2 (*RUNX2*); collagen, type I, alpha 1 (*COL1A1*); and liver/bone/kidney alkaline phosphatase (*ALPL*). Our results support the use of hypothermic storage with PBS to preserve key characteristics of tissue‐engineered human bone grafts.

## Materials and methods

### Engineering bone grafts

Human bone grafts (*n* = 18) were engineered as previously described.^26^, ^27^, ^29^ Briefly, human iPSC‐derived mesenchymal progenitor cells (line 1013A) at passage 6 were plated onto gelatin‐coated plasticware and expanded in medium consisting of high‐glucose KnockOut Dulbecco's modified Eagle's medium (DMEM; Gibco) supplemented with 20% (v/v) HyClone™ fetal bovine serum (FBS; GE Healthcare Life Sciences), fibroblast growth factor basic (1 ng/mL; Invitrogen), nonessential amino acids (0.1 mM; Gibco), glutaMAX (2 mM; Gibco), beta‐mercaptoethanol (0.1 mM; Gibco), and antibiotic‐antimycotic (100 U/mL; Gibco). Following expansion, the cells were detached using 0.25% trypsin‐ethylenediaminetetraacetic acid (Gibco), counted using a hematocytometer, and seeded onto sterile decellularized cow bone disks (8 mm in diameter and 5 mm in height) at a density of 10^6^ cells per scaffold using a drop technique.^27^ Following seeding, the samples were cultured in an osteogenic environment consisting of high‐glucose DMEM medium supplemented with 10% (v/v) HyClone FBS (GE Healthcare Life Sciences), dexamethasone (1 µM; Sigma), beta‐glycerophosphate (10 µM; Sigma), ascorbic acid‐2‐phosphate (50 µM; Sigma, A8960), and antibiotic‐antimycotic (100 U/mL; Gibco) for 5 weeks.

### Tissue preservation

After 5 weeks of culture in an osteogenic environment, the tissue‐engineered bone grafts were stored using two different preservation methods. Briefly, samples were washed in Dulbecco's phosphate‐buffered saline (DPBS; Gibco), placed in cryovials, and stored in DPBS (Gibco) at 4 °C or in Synth‐a‐Freeze at −80 °C for 48 hours. For storage in Synth‐a‐Freeze at −80 °C, cryovials were placed in a Nalgene® Mr. Frosty container to provide a critical 1 °C/min cooling rate required for optimal cryopreservation of cells. At the end of the preservation period, samples were rapidly revitalized in a water bath at 37 °C and then incubated in osteogenic medium for 1 and 24 h before analysis. Nonpreserved samples were used as controls for all analyses.

### Metabolic assay

The effect of storage on the metabolic activity of tissue‐engineered bone grafts was estimated using PrestoBlue™, a cell‐permeable resazurin‐based solution that functions as an indicator of the cell metabolic activity by using the reducing power of living cells. Before storage, as well as 1 and 24 h after revitalization, samples were treated with 1 mL of osteogenic medium containing 10% (v/v) of PrestoBlue reagent (Life Technologies), and incubated for 2 h at 37 °C. Following incubation, 200 µL aliquots of culture media were transferred to a black, clear, flat‐bottom 96‐well plate (BD Falcon™), and fluorescence was measured at 560/590 nm (excitation/emission) using a Synergy™ Mx fluorescence reader (BioTek) equipped with Gen 5 1.09 software. The metabolic activity of the cells is expressed as the intensity of measured fluorescence relative to values measured before treatment.

### Live/dead assay

The effect of storage on the viability of tissue‐engineered bone grafts was studied using the LIVE/DEAD assay (Thermo Scientific). Briefly, before preservation, as well as 1 and 24 h after revitalization, samples were cut longitudinally in half, washed in DPBS (Gibco), and incubated with a solution of calcein AM (2 mM) and ethidium bromide (4 mM) in DPBS (Gibco) for 1 h at 37 °C in the dark. Following incubation, the samples were washed in DPBS (Gibco), and then placed in RPMI medium (without red phenol; Lonza) for imaging. Fluorescence micrographs of the center of the scaffold were taken with an Olympus IX71 microscope and combined into mosaics using ImageJ (National Institutes of Health) software equipped with the MosaicJ and TurboReg plugins. Confocal images were taken with the Axiovert 200M microscope (Carl Zeiss AG) mounted with LSM 5 Pascal exciter and using the LSM 5 Pascal software with defined settings. Quantification of dead (red) and live (green) cells was conducted in ImageJ (National Institutes of Health) using the open source image processing package Fiji. Briefly, confocal composite images were split into two channels, and the area covered by dead and live cells was detected with pixel values set to 61 and 115, respectively. Data are shown as the percentage area of dead cells per area of live cells.

### Histology

Tissue formation and the effects of storage on the integrity of the extracellular matrix were studied via histological analysis. Before storage, as well as 1 and 24 h after revitalization, samples were cut longitudinally in half and washed in DPBS (Gibco) at room temperature for 5 minutes. Samples were then fixed in 4% (v/v) paraformaldehyde in PBS (Santa Cruz Biotechnology) at 4 °C for 2 days, decalcified in Immunocal™ (Decal Chemical Corporation) at 4 °C for 4 days, and then dehydrated through graded concentrations of ethanol prior to paraffin embedding. Samples were finally cut into 5 µm–thick sections, mounted on charged glass slides, and stained with hematoxylin and eosin and with Sirius Red. Sections stained with Sirius Red were observed under infrared light^30^ using an Axiovert 200M confocal microscope (Carl Zeiss AG) mounted with an LSM 5 Pascal exciter and using the LSM 5 Pascal software with defined settings.

### Immunohistochemistry

The effects of storage on the viability and composition of tissue‐engineered bone grafts were studied via immunohistochemical analysis. Briefly, 5 µm–thick sections (prepared as above) were deparaffinized by heating at 60 °C for 30 min, followed by incubation in CitriSolv® (twice for 5 min; Decon Labs, Inc), rehydrated with a graded series of ethanol washes (twice 100%, 95%, 70%, and 50%; each for 2 min), incubated in deionized H_2_O (three times for 2 min), and washed in DPBS (Gibco) for 5 minutes. The sections were then incubated in citrate buffer (pH 6) at 90 °C for 30 min for antigen retrieval, washed in deionized H_2_O for 5 min, and incubated with 3% (v/v) H_2_O_2_ in methanol for 30 min to block endogenous peroxidase activity. Following a wash in DPBS (Gibco) for 5 min, sections were incubated with 1% (v/v) normal horse serum (Vectastain ABC kit Elite, #PK‐6200 Universal) in DPBS (Gibco) to block nonspecific binding, and stained at 4 °C overnight in a humidified chamber with primary antibodies (all diluted 1:500 in DPBS) against cleaved caspase‐3 (Cell Signaling Technology, #9664), osteopontin (Millipore, #AB1870), osteocalcin (Millipore, #AB10911), and bone sialoprotein (Millipore, #AB1854). Specific antigen detection was performed using biotinylated secondary antibody and biotin/avidin‐based peroxidase complex (Vectastain® Elite® ABC Kit) diluted in DPBS (Gibco) according to manufacturer's instructions, and followed by incubation with the peroxidase substrate 3,3’‐diaminobenzidine for 5 min (Vector DAB kit). Sections were then counterstained with hematoxylin (Richard‐Allan Scientific), dehydrated with a graded series of ethanol washes (50%, 70%, 95%, and twice 100%; each for 2 min), incubated with CitriSolv (twice for 5 min), dipped into xylene, and sealed with a coverslip (Fisherbrand) using Permount mounting medium (Fisher Chemicals Scientific).

Negative controls were performed following the same procedure but omitting either the primary or secondary antibody incubation.

Quantification of cells positive for cleaved caspase‐3 was conducted in ImageJ (National Institutes of Health) using the open source image processing package Fiji. Briefly, immunohistochemical images were color deconvoluted into hematoxylin, DAB, and background images. DAB images were then analyzed to quantify the area covered by cells positive for cleaved caspase‐3 by applying a global thresholding with pixel values set to 63. Data are shown as percentage of cleaved caspase‐3–positive cells per total tissue area.

### Real‐time polymerase chain reaction

The effect of storage on the expression of genes involved in osteogenesis was analyzed via real‐time PCR. Briefly, before preservation and 1 h after revitalization, samples were lysed in TRIzol™ buffer (Qiagen) and total RNA was isolated using the RNeasy Mini Kit (Qiagen). Following quantification, 500 ng of RNA was reverse transcribed with random hexamers using the GoScript™ Reverse Transcription System (Promega) according to the manufacturer's protocol. The expression of *RUNX2* (Hs00231692_m1), *COL1A1* (Hs00164004_m1), *ALPL* (Hs01029144_m1), and the housekeeping gene glyceraldehyde 3‐phosphate dehydrogenase (*GAPDH*; Hs02758991_g1) was analyzed in a 20 µL volume reaction using the TaqMan™ Universal PCR Master Mix and TaqMan Gene Expression Assay (Applied Biosystems) in a StepOnePlus PCR System cycler (Applied Biosystems). The expression levels of the target genes are expressed as normalized to the expression level of GAPDH.

### Image processing and generation

Image levels and backgrounds were adjusted in Adobe Photoshop CC (Adobe Systems Incorporated) to improve viewing. Images were assembled into figure panels using Adobe Illustrator CC (Adobe Systems Incorporated).

### Statistical analysis

Statistical analysis was conducted using GraphPad Prism 6 version 6.0e. Repeated measures analysis of variance (ANOVA) with a Bonferroni post‐hoc test was used to compare the metabolic activity of samples before and after preservation. An unpaired *t*‐test was used to compare the metabolic activity of samples treated using different preservation methods, as well as the percentage of dead and apoptotic cells between control and the two treatment groups. A one‐way ANOVA for multiple comparisons with Bonferroni post‐hoc test was used to compare gene expression between control and treatment groups. Results are shown as means ± standard deviations. Differences were considered statistically significant when the *P* value was less than 0.05.

## Results

### Tissue viability

**Figure 1:**
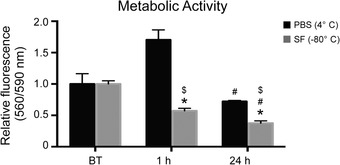
Metabolic activity. PrestoBlue analysis showing the metabolic activity of tissue‐engineered bone grafts before treatment (BT), as well as 1 and 24 h after storage in phosphate‐buffered saline solution (PBS) at 4 °C and in Synth‐a‐Freeze (SF) at −80 °C. Data represent means ± standard deviations (*n* = 3, *P* < 0.05; ^*^ denotes a significant difference compared with samples before preservation, # denotes a significant difference between time points for the same treatment, and $ denotes a significant difference between treatments for the same time point).

**Figure 2:**
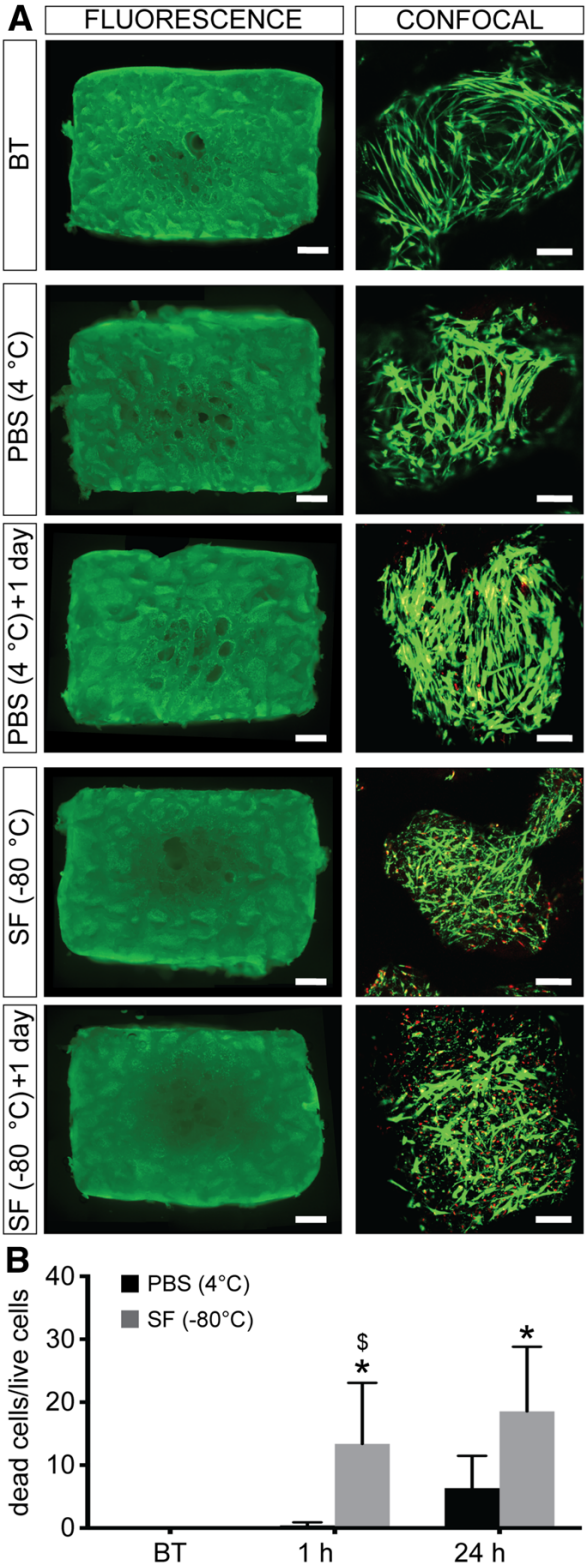
Tissue viability. (A) Epifluorescence (mosaic) and confocal images of tissue‐engineered bone grafts before treatment (BT), as well as 1 and 24 h after storage in phosphate‐buffered saline solution (PBS) at 4 °C and in Synth‐a‐Freeze (SF) at −80 °C. Viable cells stain green and dead cells stain red. Scale bars: 1 mm and 100 µm, respectively. (B) Quantification of dead cells in confocal images of samples before treatment (BT), as well as 1 and 24 h after storage in phosphate‐buffered saline solution (PBS) at 4 °C and in Synth‐a‐Freeze (SF) at −80 °C. Data represent means ± standard deviations (*n* = 3, *P* < 0.05; ^*^ denotes a significant difference compared with samples before preservation, and $ denotes a significant difference between treatments for the same time point).

**Figure 3:**
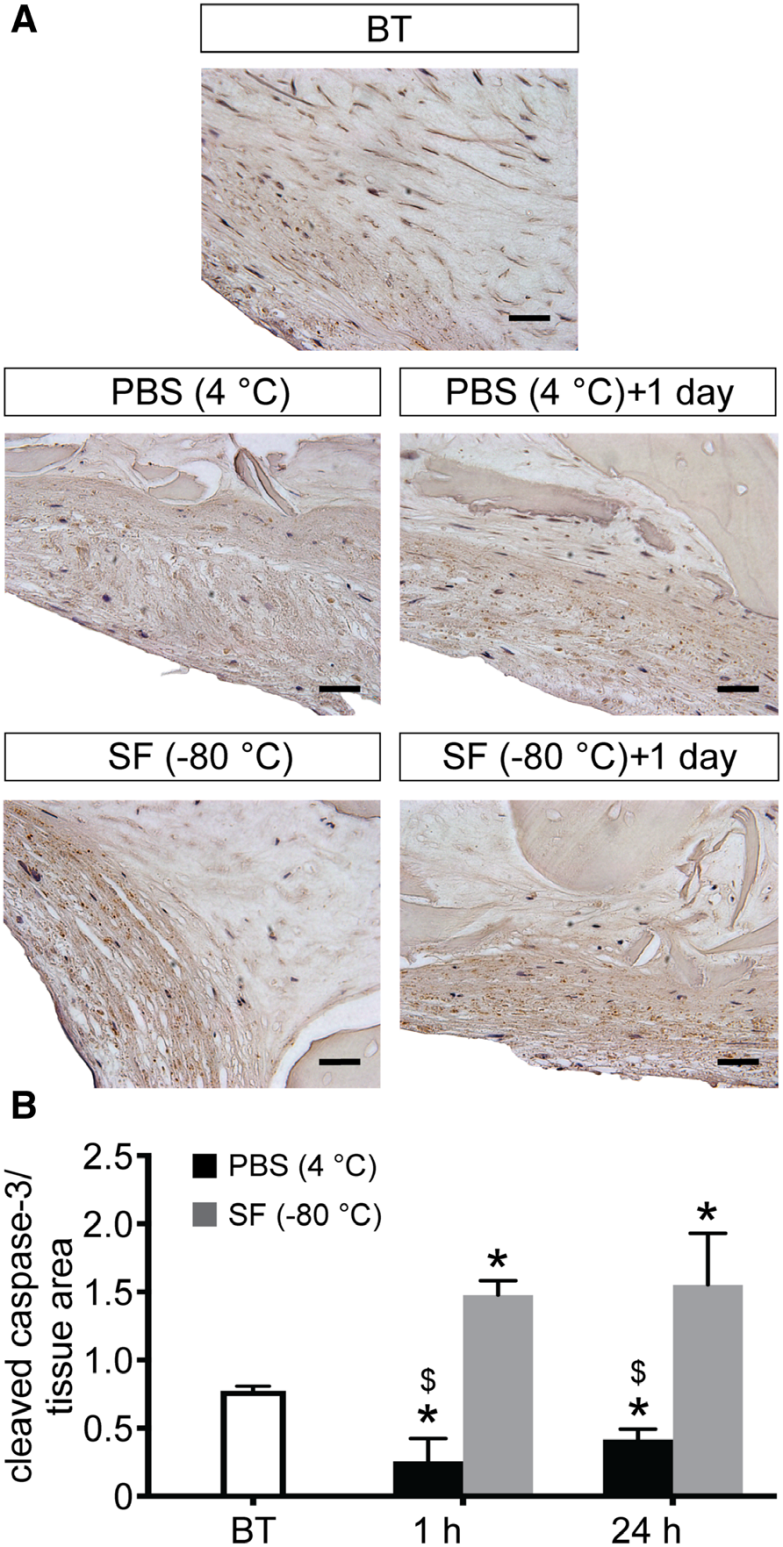
Apoptotic cells. (A) Immunohistochemical assessment of active caspase‐3 in tissue‐engineered bone grafts before treatment (BT), as well as 1 and 24 h after storage in phosphate‐buffered saline solution (PBS) at 4 °C and in Synth‐a‐Freeze (SF) at −80 °C. Scale bar: 20 µm. (B) Quantification of apoptotic cells in immunohistochemical micrographs of samples before treatment (BT), as well as 1 and 24 h after storage in phosphate‐buffered saline solution (PBS) at 4 °C and in Synth‐a‐Freeze (SF) at −80 °C. Data represent means ± standard deviations (*n* = 3, *P* < 0.05; ^*^ denotes a significant difference compared with samples before preservation, and $ denotes a significant difference between treatments for the same time point).

The effects of tissue storage on cell function and viability were estimated using a combination of biochemical assays and immunohistochemistry. PrestoBlue assay data reveal an increase in metabolic activity 1 h after revitalization for tissue samples stored in PBS at 4 °C (Fig. 1), although this is not statistically significant. However, following 24 h incubation at 37 °C, the metabolic activity of the tissue samples decreases and reaches levels similar to those observed for control samples (i.e., before treatment). On the other hand, samples stored in Synth‐a‐Freeze at −80 °C display a significant decrease in metabolic activity both 1 and 24 h after revitalization. Interestingly, the metabolic activity is significantly lower when samples are stored at cryogenic temperature in Synth‐a‐Freeze compared with hypothermic temperature in PBS, indicating a more harmful effect of this condition on tissue function. Live/dead fluorescence staining of samples, before and after storage, reveals increased cell death following preservation in PBS at 4 °C and in Synth‐a‐Freeze at −80 °C (Fig. 2). Interestingly, for samples stored at 4 °C, the number of dead cells seems to increase 24 h following revitalization compared with control samples. By contrast, samples preserved in Synth‐a‐Freeze at −80 °C contain far more dead cells (red) even 1 h after revitalization, which increases even further 24 h after revitalization. Concomitant with an increase in the number of dead cells, live cells (green) appear more pixelated, perhaps indicating the damage of the cell membrane and leakage of the fluorescent dye from dying cells. In fact, immunohistochemical analysis of paraffin‐embedded tissue sections (Fig. 3 and Fig. S1, online only) shows the increased presence of cleaved caspase‐3 when samples are stored in Synth‐a‐Freeze at −80 °C, compared with samples before treatment or samples preserved in PBS at 4 °C, suggesting that storage at cryogenic temperatures can trigger apoptosis and adversely affect tissue viability. Regardless of the storage method, however, samples show increased levels of cleaved caspase‐3 24 h after revitalization, although the positive staining (small dark brown dots) is much higher in samples stored in Synth‐a‐Freeze at −80 °C. This may indicate that apoptotic processes are less detectable early after revitalization, yet that they have in fact been initiated and their rate of progression depends on the preservation method.

### Tissue formation, composition, and structure

**Figure 4:**
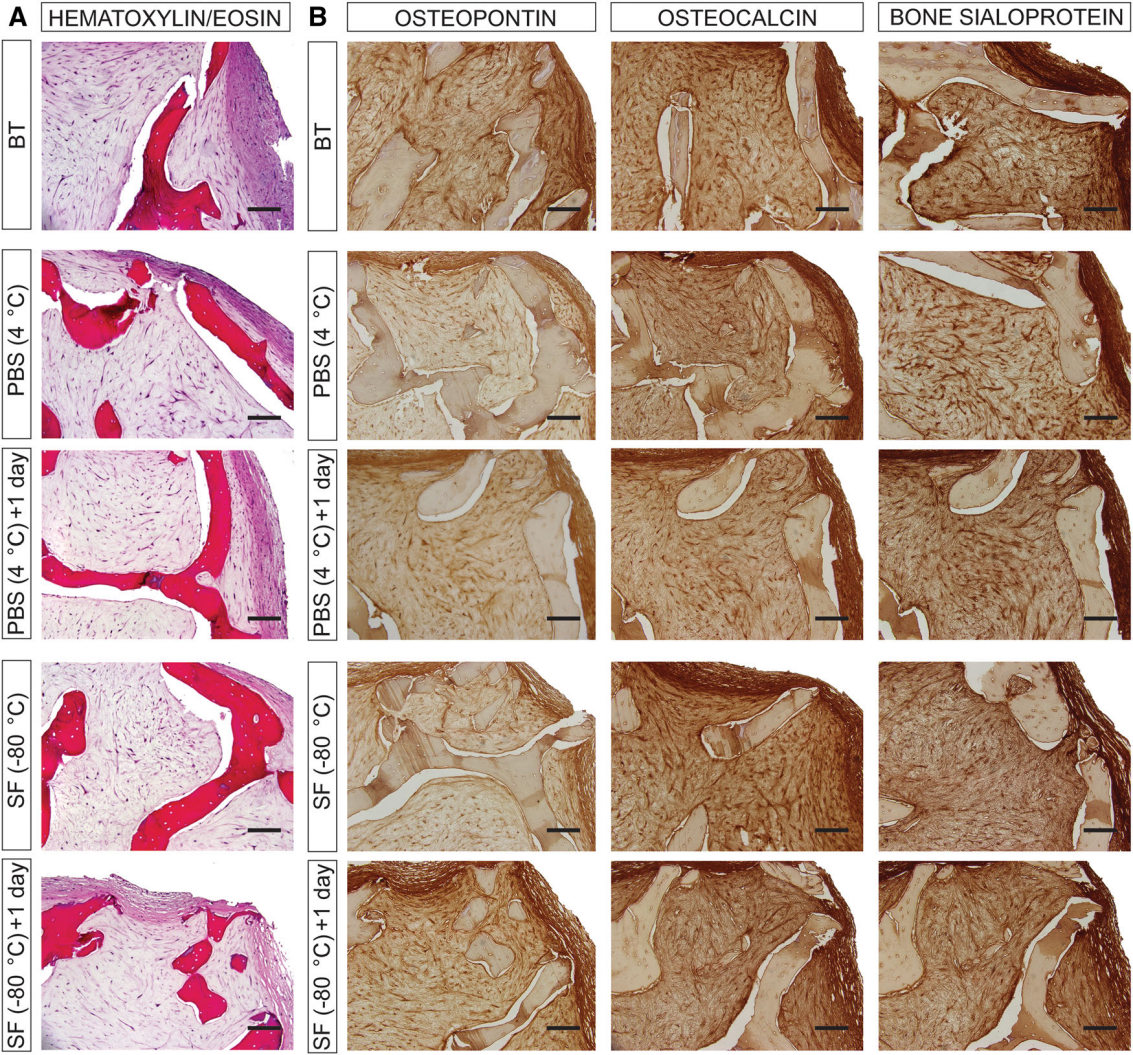
Tissue formation and composition. (A) Histological assessment of tissue‐engineered bone grafts before treatment (BT), as well as 1 and 24 h after storage in phosphate‐buffered saline solution (PBS) at 4 °C and in Synth‐a‐Freeze (SF) at −80 °C. Samples are stained with hematoxylin/eosin, which stains cell nuclei blue and the extracellular matrix pink. Scale bar: 100 µm. (B) Immunohistochemical assessment of tissue‐engineered bone grafts before treatment (BT), and 1 and 24 h after preservation in phosphate‐buffered saline solution (PBS) at 4 °C and in Synth‐a‐Freeze (SF) at −80 °C. Samples are stained for osteocalcin, osteopontin, and bone sialoprotein (brown). Scale bar: 100 µm.

**Figure 5:**
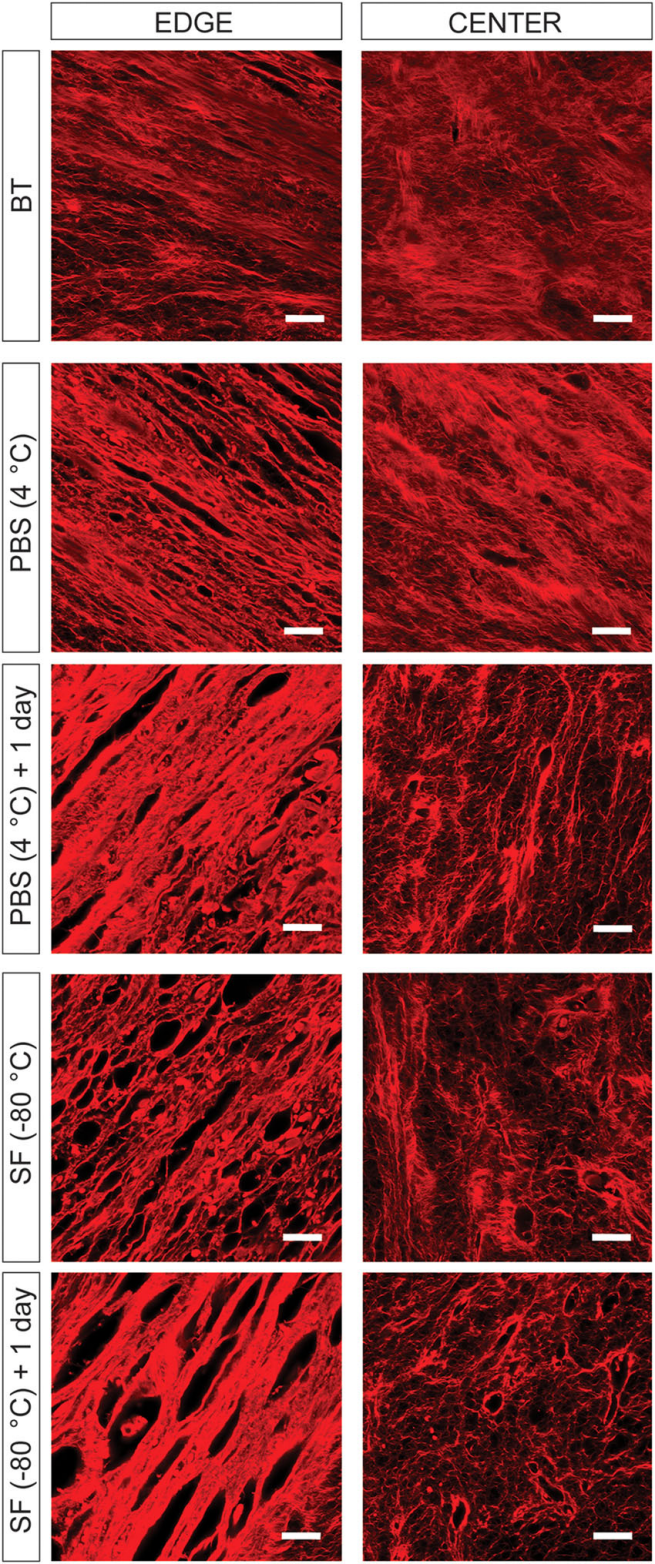
Extracellular matrix integrity. Confocal images showing the organization of collagen fibers in tissue‐engineered bone grafts before treatment (BT), as well as 1 and 24 h after storage in phosphate‐buffered saline solution (PBS) at 4 °C and in Synth‐a‐Freeze (SF) at −80 °C. Samples are stained with Sirius Red, which stains collagen fibers in Red. Scale bar: 10 µm.

The effects of storage on the quality and integrity of the extracellular matrix of tissue‐engineered bone grafts were studied through histological and immunohistochemical investigation. The analysis demonstrates the formation of compact tissue in between the scaffold trabeculae that stains positive for the bone‐specific proteins osteopontin, osteocalcin, and bone sialoprotein (Fig. 4 and Fig. S2, online only). Interestingly, no differences in tissue content and composition are observed when comparing tissue‐engineered bone grafts stored in PBS at 4 °C and in Synth‐a‐Freeze at −80 °C with control samples (i.e., before treatment), indicating that the methods tested in this study do not alter the conformation and spatial distribution of the characteristic proteins constituting the extracellular matrix of the bone tissue. On the other hand, closer examination of the extracellular matrix under infrared light reveals that storage affects the integrity/organization of the collagen fibers (Fig. 5), an effect that is more pronounced when samples are stored in Synth‐a‐Freeze at −80 °C compared with hypothermic temperature in PBS, especially 24 h after revitalization. The results show structural alteration of the collagen fibers both at the edge and center of the tissue‐engineered bone grafts. At the edge of the tissue, where the collagen fibers are denser and aligned in parallel, it is possible to observe thickening and structural separation, a phenomenon that worsens 24 h after revitalization. At the center of the tissue instead, where the collagen fibers are less dense and randomly oriented, preservation results in the formation of empty pockets in the fabric of the extracellular matrix.

### Expression of osteogenic genes

**Figure 6:**
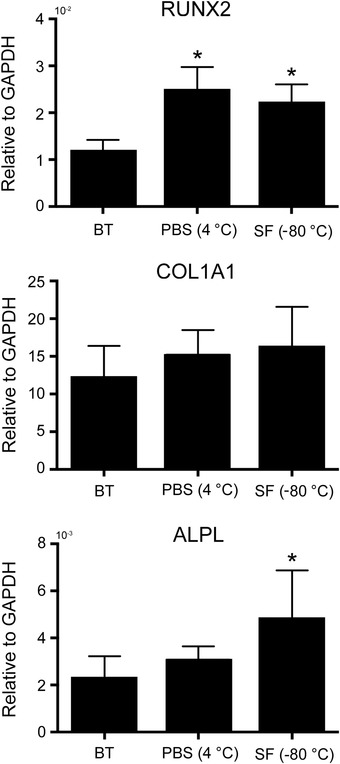
Gene expression. Real‐time polymerase chain reaction data showing the expression of *RUNX2*, *COL1A1*, and *ALPL* in tissue‐engineered bone grafts before treatment (BT), as well as 1 and 24 h after storage in phosphate‐buffered saline solution (PBS) at 4 °C and in Synth‐a‐Freeze (SF) at −80 °C. Data represent means ± SD (*n* = 3, *P* < 0.05; ^*^ denotes a significant difference compared with samples before preservation).

The effects of preservation on the functionality of tissue‐engineered bone grafts were studied by measuring the expression of the osteogenic genes *RUNX2*, *COL1A1*, and *ALPL*. Real‐time PCR analysis (Fig. 6) shows that preservation does not affect negatively the expression of these genes. On the other hand, the preservation methods tested in this study lead to the upregulation of *RUNX2* and *ALPL*. While *RUNX2* expression increases significantly when samples are preserved in PBS at 4 °C and in Synth‐a‐Freeze at −80 °C, ALPL expression significantly increases only when samples are preserved in Synth‐a‐Freeze at −80 °C.

## Discussion

Having available adequate amounts of personalized tissues that can be distributed and delivered to patients represents a paradigm shift in medicine that will revolutionize the way medical care is provided. To support clinical translation of engineered tissues, development and testing of preservation methods that minimize damage and increase the shelf life of these products is crucial to foster research and facilitate the transition from bench to bedside. Typically, cells and tissues are preserved at hypothermic or cryogenic temperature in physiological or cryoprotectant‐containing solutions that mitigate the biophysical effects associated with the cooling, freezing, and warming process. Cells, in particular, display good tolerance to freezing using available preservation solutions, and can be maintained viable for about 1 week at 4 °C in PBS.^3^ On the other hand, engineered tissues are more sensitive than individual cells to preservation, and the biophysical effects of hypothermic and cryogenic storage vary markedly based on the size and type of tissue and in relation to their specific structural organization. Over the past years, many efforts have been made to develop and test preservation protocols and solutions, and to study the effects of hypothermic and cryogenic temperatures on tissue‐engineered products, including neural tissue, cartilage, mucosa, skin, vascular grafts, and so on.^5^, ^9^, ^31^, ^32^, ^33^, ^34^, ^35^ However, no knowledge exists on the effects of these conditions on human bone engineered from human iPSCs. These cells can be derived from every patient, produced in clinically sufficient numbers, and coaxed to become any cell type constituting the human body.^25^ The ability to store and/or bank large amounts of personalized bone will facilitate a tissue engineering approach toward personalized orthopedic reconstructions. In this study, we tested the effects of storage in PBS at 4 °C or in Synth‐a‐Freeze at −80 °C on the survival, structure, and function of tissue‐engineered human bone grafts. Synth‐a‐Freeze is a commercially available cryopreservation medium containing 10% dimethylsulfoxide (DMSO) intended for freezing and storing a variety of mammalian cell types, including mesenchymal stem cells. To take into consideration possible delays in storage‐induced cell death and tissue damage and more thoroughly assess the effects of storage on tissue quality, samples were analyzed 1 and 24 h after revitalization. Biochemical and immunohistochemical analysis revealed significant cell death associated with storage at cryogenic temperature, and the presence of dying cells in the newly formed tissue. Cell death likely results from the physical and chemical changes that occur during freezing and thawing,^10^, ^11^ as well as from the toxicity of the DMSO present in the Synth‐a‐Freeze solution. The results are consistent with other studies testing the effects of cryopreservation on tissue‐engineered constructs, which have observed a 25% or higher decline in tissue viability following storage in cryoprotectant‐containing formulations.^34^, ^36^ Interestingly, the decline in metabolic activity and number of viable cells continues to take place following revitalization and increases after 24 h, indicating that storage‐induced damage continues to worsen following revitalization. Additional studies of metabolic activity and tissue viability at later time points are needed to fully understand the long‐term effects of cryopreservation on tissue‐engineered bone grafts and its potential impact on therapeutic efficacy. It is important to understand whether the grafts remain viable, recover, and return to values of metabolic activity observed before storage. The presence of viable cells in engineered bone grafts can support healing by directly contributing to new bone formation and/or and by inducing host cells to form new bone via the release of trophic factors.^37^ Studies in animal models of skeletal reconstructions will clarify how cryopreservation influences the regenerative properties of the grafts. In contrast with cryopreservation, storage in PBS at 4 °C did not seem to significantly affect the viability and metabolic activity of the samples, suggesting that short‐term preservation can be attained without compromising the osteoinductive and regenerative properties of tissue‐engineered human bone grafts. Additional studies are needed to test this approach, however.

In addition to affecting tissue viability, tissue storage seemed to alter the structural organization of the collagen fibers that constitute the extracellular matrix of the grafts. The extracellular matrix interacts with cells and regulates diverse cellular functions, including proliferation, migration, and differentiation.^38^ Both storage methods tested in this study resulted in the separation of the collagen fibers and the formation of empty pockets in the extracellular matrix, and these effects were much stronger when tissues were stored at cryogenic temperature. The alignment and orientation of collagen fibers confer unique mechanical properties to biological tissues.^39^ Thus, understanding how the structural modifications of the collagen fibers associated with preservation affect the quality of tissue‐engineered bone grafts becomes essential to manufacture compliant products with optimal mechanical capabilities. It also remains to be understood whether these structural alterations elicit an adverse response in the body, which compromises graft–host interaction and the healing process following implantation.

Despite changes in the ultrastructure of the extracellular matrix, all samples were positive for the noncollagenous bone matrix glycoproteins, osteopontin osteocalcin, and bone sialoprotein, indicating that storage does not result in molecular unfolding or breakdown of proteins that regulate mineralization and may impart osteoconductive properties on tissue‐engineered bone grafts.^40^ Likewise, storage at both hypothermic and cryogenic temperature did not affect the expression of *RUNX2*, *COL1A1*, and *ALPL*, which are recognized to support osteogenesis *in vitro* and *in vivo*.^41^

Altogether, the results of the present study show that hypothermic storage allows preservation without significantly compromising viability, composition, and function of tissue‐engineered human bone grafts. It remains to be understood, however, how the separation of the collagen fibers affects the reconstructive properties of bone grafts preserved at hypothermic temperatures.

By contrast, cryogenic storage preserves neither tissue viability nor the ultrastructure, which could diminish the regenerative properties and therapeutic potential of these grafts. The extent and consequences of these effects require further investigation in clinically relevant animal models before optimal protocols and formulations can be designed for short‐ and long‐term storage of tissue grafts for personalized orthopedic applications.

## Conclusions

This study provides new insights into how hypothermic and cryogenic preservation affect the characteristics of bone grafts engineered from human iPSCs. Although further study is required, the data support the idea that personalized human bone can be preserved for distribution and, perhaps, banked as an off‐the‐shelf product for advanced skeletal reconstructions.

## Competing interests

The authors declare no competing interests.

## Supporting information


**Figure S1**. Additional data on apoptotic cells. Control sample staining negative for cleaved caspase‐3, counterstained with hematoxylin. Scale bar: 20 µm.

**Figure S2**. Additional data on tissue composition. Control sample staining negative for osteocalcin, osteopontin, and bone sialoprotein, counterstained with hematoxylin. Scale bar: 100 µm.
