# Supplementary figures and images for: Hypothermic and cryogenic preservation of tissue‐engineered human bone
Source: Ann N Y Acad Sci. 2019 Oct 31;1460(1):77–87. doi: 10.1111/nyas.14264 (PMC7027566; doi:10.1111/nyas.14264)

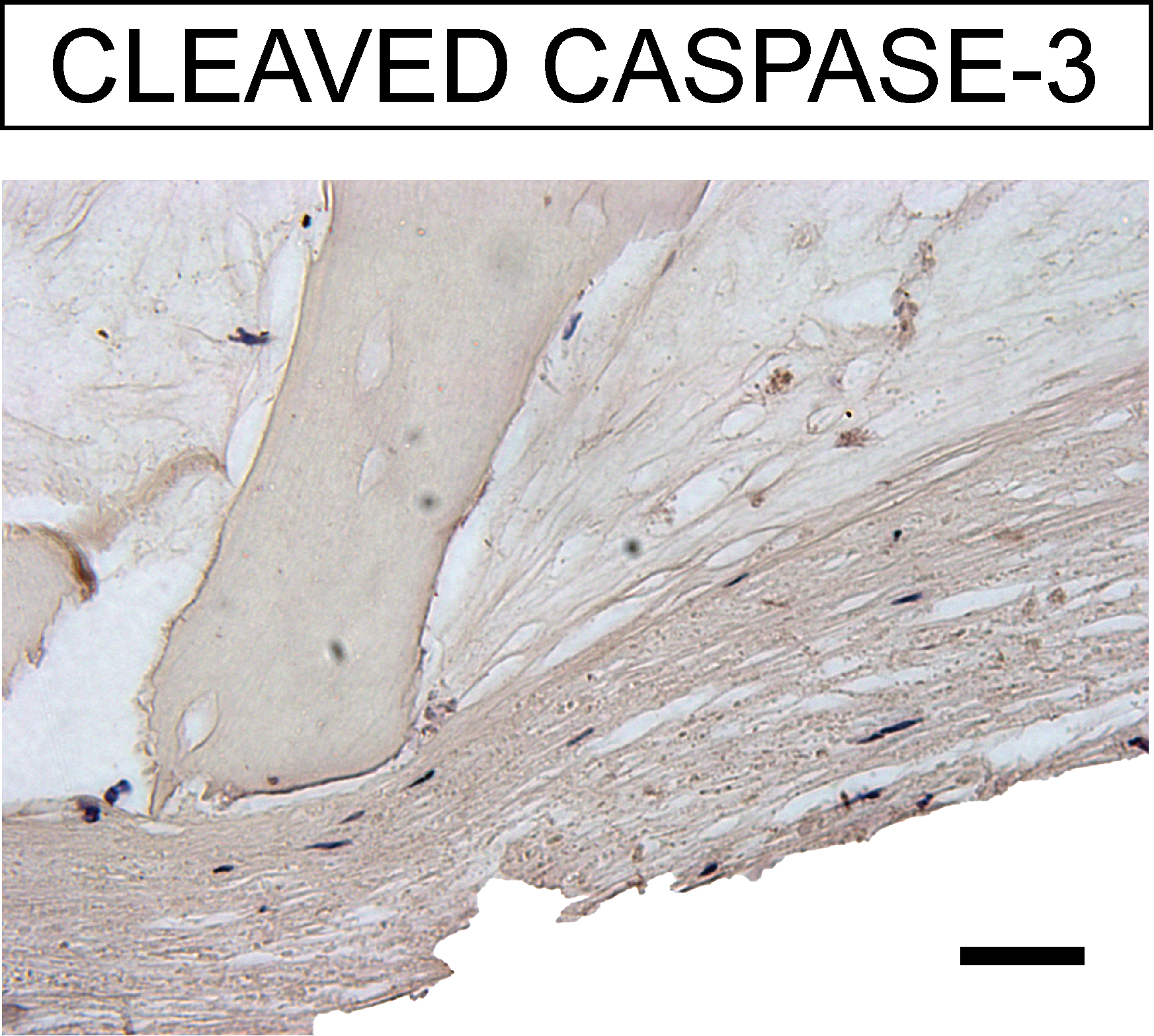

Supplement: Supplementary file 1 — Figure S1. Additional data on apoptotic cells. Control sample staining negative for cleaved caspase‐3, counterstained with hematoxylin. Scale bar: 20 µm. [file NYAS-1460-77-s001.tif]

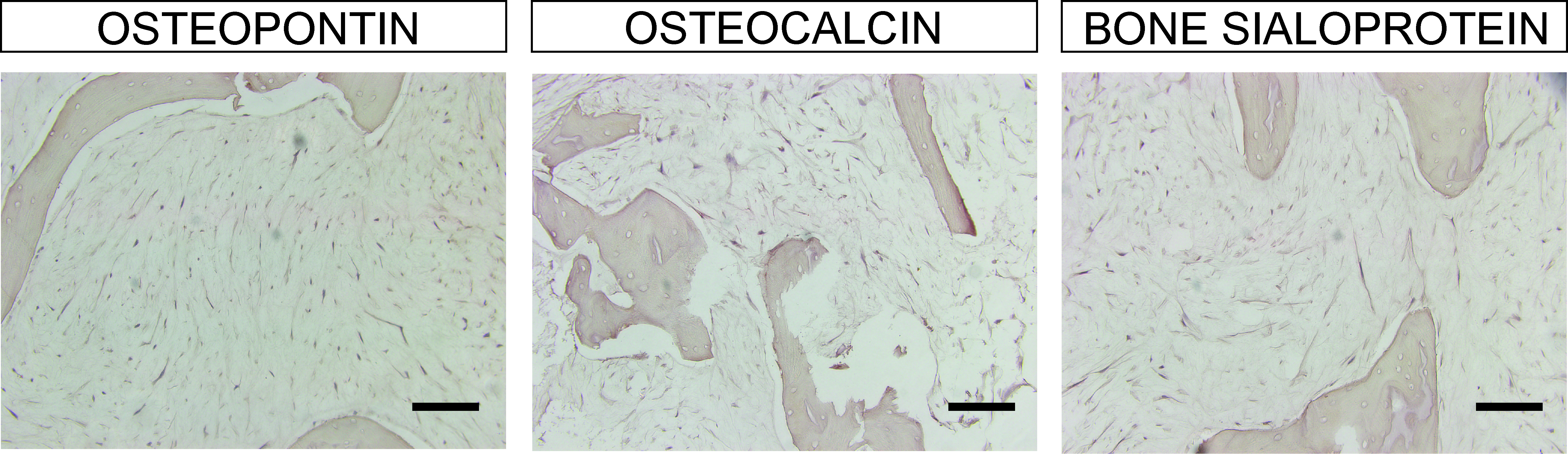

Supplement: Supplementary file 2 — Figure S2. Additional data on tissue composition. Control sample staining negative for osteocalcin, osteopontin, and bone sialoprotein, counterstained with hematoxylin. Scale bar: 100 µm. [file NYAS-1460-77-s002.tif]
